# Selecting an Individualized Treatment Approach: The Predictive Value of Erotic Stimulation and Nocturnal Erections for Efficacy of Tadalafil and Cure in Patients With Erectile Dysfunction

**DOI:** 10.3389/fendo.2022.915025

**Published:** 2022-06-29

**Authors:** Zhiwei Liu, Tao Wu, Shanjin Ma, Wei Xue, Xiaoye Jiang, Qisheng Tang, Jianjun Ma

**Affiliations:** Department of Urology, Tangdu Hospital, Air Force Medical University, Xi’an, China

**Keywords:** erectile dysfunction, tadalafil responders, cured patients, nocturnal penile tumescence and rigidity, audiovisual sexual stimulation, prognostic factors

## Abstract

**Purpose:**

This study aimed to evaluate two modes of Rigiscan for predicting tadalafil response, and to identify which Rigiscan variables are the most efficient at making these predictions.

**Methods:**

All patients received at least two rounds of nocturnal penile tumescence and rigidity (NPTR) testing and/or audiovisual sexual stimulation (AVSS), then completed the International Index of Erectile Function-5 (IIEF-5) questionnaire, followed by oral 5 mg tadalafil daily for 4 weeks. After a 4-week washout period, all respondents underwent an the IIEF-5 questionnaire again. ED patients were then categorized into tadalafil responders and tadalafil non-responders, who were then further divided into cured patients and uncured patients.

**Results:**

When predicting tadalafil responders, the area under the curve (AUC) of NPTR was superior to that of AVSS (0.68~0.84 VS 0.69~0.73), and the predicted optimal cut-off values were DOEE60≥17.75 min in NPTR, compared to other parameters regardless of AVSS or NPTR (P<0.05). When predicting which patients would be cured, the AUC of AVSS was superior to NPTR parameters (0.77~0.81 vs 0.61~0.76), and the determined best diagnostic cut-off values were DOEE≥4.125min in AVSS, compared to other parameters regardless of AVSS or NPTR (P < 0.05).

**Conclusion:**

Rigiscan was able to predict the efficacy of daily tadalafil accurately and efficiently. Its diagnostic value was at maximum when DOEE60 ≥17.75 min of NPTR in tadalafil responders and DOEE ≥ 4.125 min of AVSS in cured patients.

## Introduction

Erectile dysfunction (ED), a type of sexual dysfunction that is global (1), negatively influences self-esteem and sexual quality of life in males. Research examining erectile dysfunction has revealed that inhibiting the phosphodiesterase type 5 enzyme can increase the concentration of cyclic guanosine monophosphate (cGMP) in the penile cavernosa, resulting in the smooth muscle relaxation that is associated with penile erection (2). Four phosphodiesterase type 5 inhibitors (PDE5Is) have been approved as first-line therapy for patients with erectile dysfunction (ED) based on their efficacy and safety in the large majority of ED populations, including tadalafil, sildenafil, avanafil, and vardenafil (3, 4). Tadalafil (5 mg once per day) has a long half-life of 17.5 h, implying that it has the ability to effectively reduce PDE5 enzyme levels in most ED patients (5, 6). According to a review by Utsav et. al., once-daily tadalafil offers better scores on the International Index for erectile dysfunction- erectile function domain (IIEF-EF) than on-demand treatment (7). More recently, clinical studies for ED treatment have focused not just on male sexual functioning but also on the sexual quality of life of their female sexual partners (8). Because PDE5Is do not initiate erections and sexual stimulation is still required to aid an erection (9), some patients with ED are unable to achieve a sexually gratifying erection after taking 5 mg tadalafil once daily for a substantial amount of time. Therefore, it is necessary to determine which parameters can predict tadalafil response or treat ED.

Many studies have revealed that such a response may be predicted by the international index of erectile function-5 (IIEF-5) questionnaire scores (10), along with invasive procedures such as cavernosal measurement or penile biopsy (11) in order to prevent unnecessary healthcare costs. However, these approaches are either subjective or prone to psychological resistance in patients, leading to their withdrawal from treatment. Introduced in 1985, the RigiScan gadget has been in clinical use as a simple and non-invasive method of diagnosing and treating male impotence since its inception (12). This device has two operating modes: nocturnal penile tumescence and rigidity (NPTR) and audiovisual sexual stimulation (AVSS). The former is utilized for continuous monitoring of penile erection during sleep, as it has been hypothesized that organic variables without psychogenic factors can interfere with nocturnal erection in numerous ways (13). As a result, the severity of ED may be objectively diagnosed based on NPTR results, and the patient’s response to PDE5Is predicted (14, 15). However, most research to date has focused on sildenafil rather than daily tadalafil treatment. In comparison to NPTR, the AVSS test not only reflects penile erection, but also stimulates the patient’s sexual arousal phase while awake. However, most previous studies have employed AVSS as a screening tool for ED (16), and little research has been conducted to determine whether AVSS can predict the effect of tadalafil.

This study aimed to evaluate two modes of Rigiscan for predicting tadalafil response, and identify which Rigiscan variables are the most efficient at making these predictions.

## Materials and Methods

### Patient Population

The local ethics committee approved all operations done in this research (ethics approval number: K202201-04). Informed consent was not required for this study because of its retrospective nature.

We extracted 273 cases that fulfilled our inclusion criteria from the medical records of the department of urology at Tangdu hospital from between January 2016 and February 2021. Inclusion criteria included men with a regular sexual life; men with an ED history of over 3 months; men aged ≤ 55 years and ≥ 18 years; men with complete medical records, normal hormone levels, AVSS test results, and NPTR monitoring; and those given tadalafil 5 mg daily as described below. Exclusion criteria included patients with spinal cord injuries, concomitant neurologic disease, severe heart disease, or penile fibrosis; patients taking PDE5Is during the preceding four weeks; patients taking medicines that interfered with tadalafil efficacy; and patients with difficulty falling asleep.

### AVSS Test

Patients were separated and placed on comfortable tables in private examination rooms that were dimly lighted and quiet. A Rigiscan Plus (Gesiva Medical, Eden Prairie, MN, USA) sensor was linked to the tip and base of the penis. Before stimulation, a baseline of 15 min was obtained. The hardness and degree of tumescence of the penis were examined for 60 min while the patients wore an audiovisual headset with individually erotic video stimulation. Other forms of penile stimulation were not permitted during AVSS assessment.

### NPTR Monitoring

Patients were monitored for NPTR with RigiScan plus equipment at least twice in the sleep unit, and dates were obtained each morning. The penis was attached to the detecting device in the same manner specified for the AVSS test, and the most acceptable NPTR results were evaluated. Tea, coffee, alcohol, drugs, or smoking were all prohibited during the test to ensure sleep quality.

### Tadalafil Administration and Clinical Evaluation

After performing an AVSS test and NPTR monitoring, all patients were requested to complete an IIEF-5 questionnaire. IIFE-5 patient scores were used to identify the severity of ED at the outset: mild (IIEF-5 12–21), moderate (IIEF-5 8–11), or severe (IIEF-5 ≤7). Then, for 4 weeks, 5 mg of tadalafil was administered once daily at the same time each day. After a 4-week washout period, patients were classified as tadalafil responders or non-responders based on their progress from baseline to the endpoint. IIEF-5 scoring was as follows: Improvement in IIEF-5 scores from baseline to endpoint of 2 points (mild ED), 5 points (moderate ED), or 7 points (severe ED) indicates that tadalafil treatment is effective otherwise ineffective (17). Afterward, the patients were further divided into two groups: cured patients (IIEF-5≥22) and uncured patients (IIEF-5 < 22).

### Statistical Analyses

Sample size for the different groups was calculated using PASS 15 software. The student’s t-test and the Mann–Whitney test were used to investigate the difference for continuous variants. The Kolmogorov–Smirnov test was used prior to statistical analysis to confirm the normal distribution of variables. Categorical variants were evaluated using the chi-square test. The receiver operator characteristic (ROC) curve evaluated the diagnostic accuracy and area under the curve (AUC) of all Rigiscan parameters. The Youden index was used to determine the optimal cut-off values. The Z-test was used to compare different ROC curves. A P < 0.05 was considered statistically significant.

## Results

### Patient Demographics and Baseline Characteristics

This study included 273 ED patients. Prior to tadalafil delivery, 226 patients were subjected to an AVSS test, and 204 patients were subjected to NPTR monitoring, with a total of 157 patients completing both tests. We compared age, married status, presence of children, history of smoking and alcohol use, premature ejaculation, ED duration, basic IIEF-5 scores, endpoint IIEF-5 scores, the number of tadalafil responders, and cured cases patients between the AVSS and NPTR groups. There were no statistically significant differences found (P>0.05, **Table 1**).

**Table 1:** Subject demographic and baseline characteristics.

Variables	AVSS group (n=226)	NPTR group (n=204)	P-value
^*^Age (years)	31.00 (11.25)	32.00 (12.00)	P=0.594
Married status, n (%)	182 (80.5)	162 (79.4)	P=0.772
Presence of children, n (%)	109 (48.2)	106 (52.0)	P=0.440
Smoking, n (%)	95 (42.0)	88 (43.1)	P=0.818
Alcohol use, n (%)	48 (21.2)	37 (18.1)	P=0.420
Premature ejaculation n (%)	55 (24.8)	45 (22.1)	P=0.506
^*^ED duration (month)	10.50 (15.00)	10.00 (15.00)	P=0.791
*Basic IIEF-5 scores	13.00 (5.00)	13.00 (6.00)	P=0.645
*Endpoint IIEF-5 scores	19.00 (6.25)	19.00 (8.00)	P=0.813
Tadalafil Responders, n (%)	184 (81.4)	158 (77.5)	P=0.309
Cured patients, n (%)	81(35.8)	84 (41.2)	P=0.256

*Data are shown as median (interquartile range).

### Rigiscan Parameters in Groups With Different Tadalafil Efficacy

The duration of erectile episodes (DOEE), duration of erectile episodes over 60% (DOEE60), average event rigidity (AER) of the tip and base, and tumescence of tip and base *via* AVSS were all higher in tadalafil responders (**184**, 81.4%) compared to non-responders (**42**, 18.6%; P < 0.05, **Supplementary Table S1**), and all parameters *via* AVSS in cured patients (**81,** 35.8%**)** were higher than non-cured patient (**145**, 64.2%; P<0.05, **Supplementary Table S3**). Similarly, excluding the aforementioned parameters, times of erectile episodes over 60% (TOEE60) and times of total tumescence *via* NPTR were higher in tadalafil responders (**158**, 77.5%) than in tadalafil non-responders (**46**, 22.5%), with statistical significance (P<0.05, **Supplementary Table S2**). The same results applied to the comparison between cured patients (**84**, 41.2%) and uncured patients (**120**, 58.8%) *via* NPTR (P < 0.05, **Supplementary Table S4**).

### Diagnostic Accuracy and Optimal Cut-Off Values of Rigiscan Parameters for Tadalafil Responders’ Predictions

**Figure 1:**
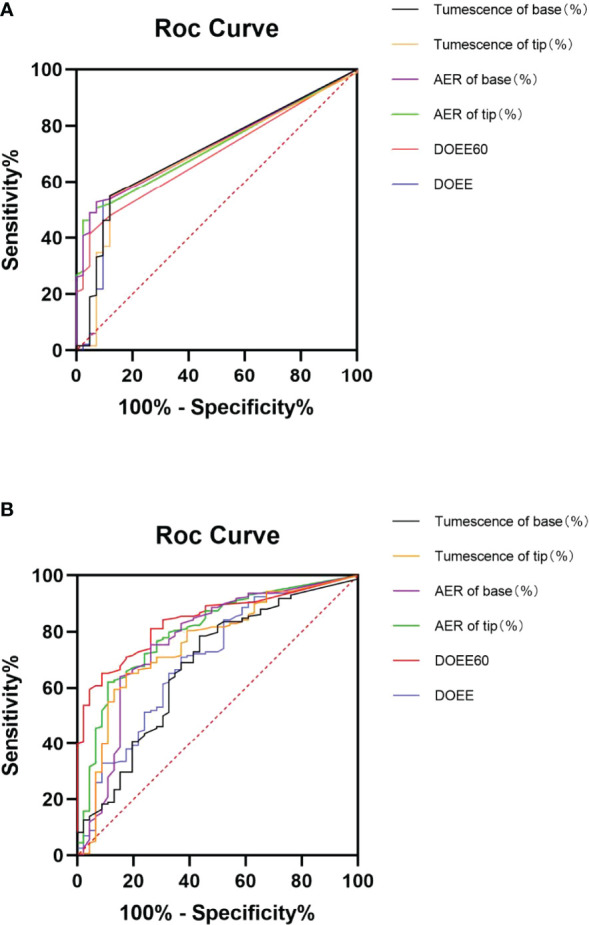
ROC curve of AVSS **(A)** and NPTR **(B)** parameters for predicting tadalafil responders. Six NPTR parameters, compared to AVSS parameters, may reliably identify tadalafil responders following 4-week 5 mg daily dosing, with significantly greater accuracy in DOEE60 in NPTR than other parameters. AER is for average event rigidity; DOEE stands for a duration of erectile episodes; ROC stands for receiver operator characteristic; AVSS stands for audiovisual sexual stimulation, and NPTR stands for nocturnal penile tumescence and rigidity.

The AUC of NPTR parameters was higher than that of AVSS parameters (0.68~0.84 VS 0.69~0.73), implying that NPTR could more accurately predict tadalafil responders than AVSS following a 4-week 5 mg daily dose. Furthermore, the DOEE60 *via* NPTR exhibited a higher AUC than AVSS index (0.84 VS 0.70, P<0.05, **Table 2** and **Figure 1**).

**Table 2:** Comparison between AVSS group and NPTR group *via* AUC in tadalafil responders prediction.

Parameters	AVSS group (n=226)	NPTR group (n=204)	P-value
DOEE (min)	0.70 (0.62~0.79)	0.70 (0.61~0.79)	P=0.963
DOEE60 (min)	0.70 (0.62~0.77)	0.84 (0.79~0.90)	P=0.002
AER of tip (%)	0.72 (0.66~0.79)	0.80 (0.73~0.88)	P=0.120
AER of base (%)	0.73 (0.66~0.80)	0.77 (0.68~0.85)	P=0.511
ΔTumescence of tip (%)	0.69 (0.61~0.78)	0.76 (0.67~0.84)	P=0.301
ΔTumescence of base (%)	0.71(0.63~0.79)	0.68 (0.58~0.77)	P=0.628

Data are shown as AUC (95% confidential interval). ΔTumescence = (increased or maximum tumescence - minimum tumescence)/minimum tumescence.

When the Youden index was used to determine the cut-off values of various Rigiscan characteristics for predicting tadalafil responders, all parameters were determined to be more specific than sensitive, as indicated in **Table 3**. The predicted best diagnostic cut-off values in NPTR parameters were DOEE60 ≥ 17.75 min instead of other parameters, regardless of AVSS or NPTR.

**Table 3:** Optimal cut-off values between AVSS group and NPTR group in tadalafil responders prediction.

Parameters	AVSS group (n=226)			NPTR group (n=204)		
	Sensitivity	Specificity	Cut-off values	Sensitivity	Specificity	Cut-off values
DOEE (min)	0.533	0.905	4.125	0.709	0.630	39.00
DOEE60 (min)	0.413	0.952	0.75	0.652	0.913	17.75
AER of tip (%)	0.489	0.952	12.5	0.620	0.891	46.50
AER of base (%)	0.527	0.929	3.00	0.753	0.739	41.50
ΔTumescence of tip (%)	0.543	0.881	2.00	0.652	0.826	24.61
ΔTumescence of base (%)	0.549	0.881	5.13	0.785	0.565	26.42

ΔTumescence, (increased or maximum tumescence - minimum tumescence)/minimum tumescence.

### Diagnostic Accuracy and Optimal Cut-Off Values of Rigiscan Parameters for Cured Patients’ Predictions

**Figure 2:**
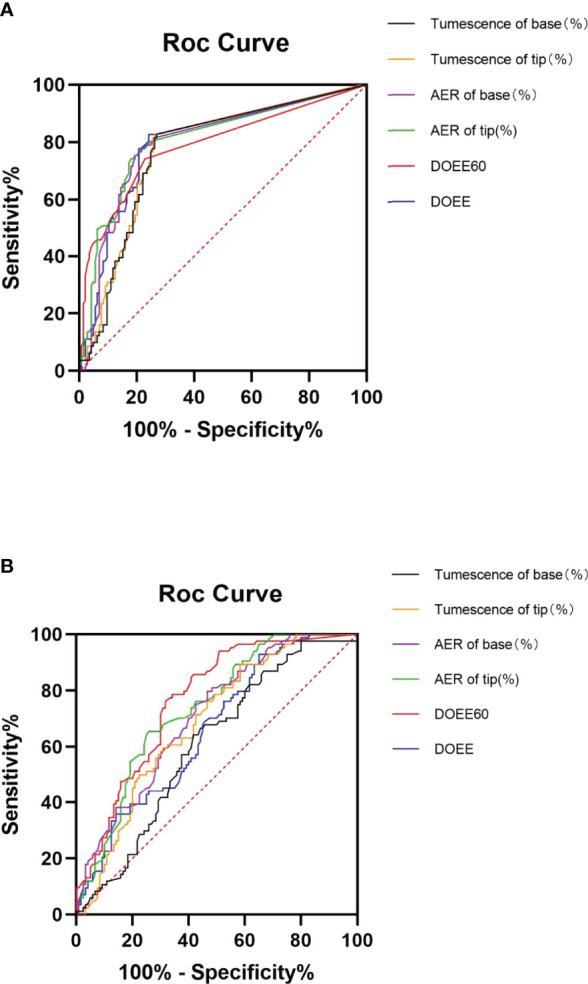
ROC curves of AVSS **(A)** and NPTR **(B)** parameters for cured patient prediction. Compared to NPTR parameters, six AVSS measures could reliably predict cured patients after four weeks of daily 5 mg tadalafil therapy, with significantly greater accuracy in DOEE and base tumescence in AVSS than other parameters. AER is for average event rigidity; DOEE stands for a duration of erectile episodes; ROC stands for receiver operator characteristic; AVSS stands for audiovisual sexual stimulation, and NPTR stands for nocturnal penile tumescence and rigidity.

After 4 weeks of 5 mg tadalafil daily administration, AVSS showed superiority to NPTR in predicting cured patients (0.77~0.81 vs 0.61~0.76). More specifically, DOEE and ΔTumescence of base *via* AVSS had the higher AUC than the index of NPTR (0.80 VS 0.66, P<0.05; 0.77 VS 0.61, P<0.05; **Table 4** and **Figure 2**).

**Table 4:** Comparison between AVSS group and NPTR group *via* AUC in cured patients predictions.

Parameters	AVSS group (n=226)	NPTR group (n=204)	P-value
DOEE (min)	0.80 (0.74~0.86)	0.66 (0.58~0.73)	P=0.004
DOEE60 (min)	0.79 (0.72~0.86)	0.76 (0.70~0.83)	P=0.601
AER of tip (%)	0.81 (0.75~0.87)	0.74 (0.67~0.80)	P=0.124
AER of base (%)	0.80 (0.74~0.86)	0.71 (0.64~0.78)	P=0.062
ΔTumescence of tip (%)	0.77 (0.71~0.84)	0.69 (0.61~0.76)	P=0.069
ΔTumescence of base (%)	0.77 (0.70~0.83)	0.61 (0.53~0.68)	P=0.002

Data are shown as AUC (95% confidential interval). ΔTumescence = (increased or maximum tumescence - minimum tumescence)/minimum tumescence.

When the Youden index was used to find the best cut-off values for several Rigiscan metrics in predicting cured patients, other parameters aside from AER of tip and base, were more sensitive than specific as demonstrated in **Table 5**. The predicted ideal diagnostic cut-off values in AVSS parameters were DOEE ≥ 4.125 min or ΔTumescence of base ≥ 5.13%, in contrast to other parameters irrespective of AVSS or NPTR.

**Table 5:** Optimal cut-off values between AVSS group and NPTR group in cured patients predictions.

Parameters	AVSS group (n=226)			NPTR group (n=204)		
	Sensitivity	Specificity	Cut-off values	Sensitivity	Specificity	Cut-off values
DOEE (min)	0.827	0.759	4.125	0.929	0.350	21.13
DOEE60 (min)	0.741	0.772	0.250	0.762	0.683	19.25
AER of tip (%)	0.778	0.786	8.00	0.655	0.742	51.50
AER of base (%)	0.753	0.807	15.50	0.798	0.533	48.50
ΔTumescence of tip (%)	0.827	0.738	2.00	0.893	0.417	17.43
ΔTumescence of base (%)	0.827	0.731	5.13	0.643	0.583	31.88

ΔTumescence, (increased or maximum tumescence - minimum tumescence)/minimum tumescence.

## Discussion

In this study, Rigiscan was able to predict the efficacy of daily tadalafil accurately and efficiently. Although EAU (18) and AUA (19) guidelines suggest PDE5Is as first-line drugs for ED, managing the condition has remained controversial. Furthermore, treatment options for ED patients have always been speculative and lacking in individualization, resulting in dissatisfied sexual lives and a lack of confidence in ED therapy. It is common for the efficacy of therapeutic regimens to be anticipated before treatment, ranging from the simple to the invasive ones like cavernosometry to penile biopsy (11, 20). For instance, Elhanbly et al. revealed that ED duration ≤ 2.5 years and IIEF-5 score 14 could show a response to sildenafil treatment (10). In contrast, because the independent IIEF-5 was subjective, and because earlier evaluations of therapy were exclusively based on the satisfaction of patients’ sexual lives, IIEF-5 was unable to examine the recovery of patients hierarchically. Moreover, Montorsi et al. discovered in 2000 that sildenafil was able to improve nocturnal erectile function (21). According to this theory, NPTR employing the Rigiscan device can predict the difference in post-treatment effect in ED patients of varying severity. Most studies have revealed that the NPTR parameter of base or tip rigidity has a distinct advantage in predicting the efficacy of sildenafil in patients with ED, whether they are normal patients or those who have undergone previous pelvic surgery (14, 15). According to existing studies, consuming 5 mg of tadalafil for 12 weeks has curative benefits on patients with ED of varying severity (22) and has no less benefit than PDE5-Is administration on demand. However, few studies have focused on predicting long-term daily tadalafil treatment effect.

Currently, there is unanimous consensus that ED may be successfully treated, but cannot be cured with current treatment options (9) unless penile prosthesis or hormonal treatments for certain patients are employed (23, 24). In recent years, many studies have been conducted to investigate the possibility of curing disorders using long-term PDE-5 inhibitors. According to Fusco et al., tadalafil 5 mg daily may be beneficial for treating endothelial dysfunction and potentially curing ED (25). Food does not affect the efficacy of tadalafil, which can last up to 36 h (26). In our experience, we have discovered that some individuals may be able to maintain a satisfying sexual life even after discontinuing oral 5 mg daily for 4 weeks in clinical trials.

Nonetheless, whether the present ED diagnostic test can predict patient susceptibility to being cured and which factor is predictive of cure has remained unknown. According to Basson, most ED patients have long-term concerns or confusion about erectile function before ED and pinpointing the crucial point of disruption in patients is critical for guiding treatment (27). Conventional detection tools, whether invasive or non-invasive, appear only to indicate penis organic injury severity and cannot reflect whether patients’ sexual confidence and desire are compromised, making it impossible to forecast whether patients are susceptible to cure. AVSS, Rigiscan’s other detection mode, has a distinct advantage in mimicking sexual arousal in the awakened state, and its erroneous results are frequently consistent with sexual desire damage (27). However, the previous AVSS test was only valuable for screening ED patients, and its influence on treatment prediction has remained unclear.

In this study, we used the IIEF-5 questionnaire combined with Rigiscan’s two modes, AVSS and NPTR, to examine tadalafil responders and predict curability in two ED patient groups. These measurements were conducted after patients were administered oral 5 mg tadalafil daily for four weeks, which has rarely been performed in prior trials. We evaluated therapy outcomes following EAU guidelines for erectile dysfunction published in 2021 while considering patients’ self-perceived erectile function improvement as measured by the IIEF-5 questionnaire (9). The 4-week post-treatment endpoint was used for this investigation. The IIEF-5 questionnaire was used to assess the long-term efficacy of tadalafil, which had an impressive short-term effect. As indicated in **Table 1**, the baseline features of the two groups indicate that ED patients in this study had primary psychogenic ED. Compared to AVSS characteristics, we discovered that six selected NPTR parameters were able to correctly predict tadalafil responders with outstanding AUC (0.68~0.84, **Table 2**), with DOEE60 having the highest diagnostic accuracy. Furthermore, the best diagnostic cut-off parameter was determined to be DOEE60 ≥ 17.75 min of NPTR. The majority of these indicators had higher diagnostic specificity than sensitivity, consistent with prior reporting (15). According to the 2010 EAU guidelines for ED, duration of tip rigidity ≥ 60% indicates severity of organic penile damage (18), and numerous studies have indicated that PDE5-Is were more likely to take action with less organically injured corpora cavernosa.

Unlike NPTR, AVSS provides a distinct advantage in predicting cured patients (AUC 0.77~0.81 VS 0.61~0.76, **Table 4**), with DOEE ≥ 4.125 min of AVSS having the best diagnostic value. The bulk of measures had substantially higher diagnostic sensitivity than specificity, contradicting Rigiscan’s forecast on Tadalafil responders. Sexual stimulation and sexual desire, as first articulated in the 1970s, play critical roles in a couple’s sexual life (28). Although it is still debated whether ED patients can be fully healed, boosting sexual confidence and the sexual response has been advocated as an effective therapeutic technique for ED patients (29). AVSS can also stimulate sexual excitement in awake individuals by using audiovisual sexual stimulation. As a result, AVSS is more likely to help predict cured patients.

According to the findings of this study, if ED patients were given both NPTR monitoring and an AVSS test prior to treatment, andrologists would be able to determine the practicality of daily tadalafil and determine the efficacy of the therapeutic scheme. As a result, patients may be more confident in receiving therapy for erectile dysfunction, which is crucial for cure.

This study had several limitation. First, patients in this trial were not followed up for two or three months following therapy to assess their sexual levels, and there were no psychometric measures available for these subjects. Additionally the absence of a health control group limits the generalization of this finding. Despite the fact that Rigiscan was able to predict the efficacy of daily tadalafil, whether it was NPTR to tadalafil responders or AVSS to cured patients, the sample size of the current study was small, and some bias could not be eliminated.

## Conclusion

The findings of this study suggested new approaches for the diagnosis and treatment of ED. Obtaining data from Rigiscan’s two modes prior to treatment may assist clinical doctors in selecting the best-individualized treatment approach. Rigiscan may also predict the efficacy of daily tadalafil objectively and effectively. Its diagnostic value was maximum when DOEE60 ≥ 17.75min of NPTR in tadalafil responders and DOEE ≥ 4.125 min of AVSS in cured patients.

## Data Availability Statement

The raw data supporting the conclusions of this article will be made available by the authors, without undue reservation.

## Ethics Statement

The studies involving human participants were reviewed and approved by The institutional review board of Tangdu Hospital of Air Force Medical University (No.K202201-04). The ethics committee waived the requirement of written informed consent for participation.

## Author Contributions

Study concepts and design: JM; Data acquisition: ZL, TW, SM, WX, and XJ; Data analysis and interpretation: WX, XJ, and QT; Statistical analysis: ZL, TW, QT; Manuscript preparation: ZL, TW, and SM; Manuscript editing: ZL, TW, SM, and JM; Manuscript review: JM. All authors contributed to the article and approved the submitted version.

## Conflict of Interest

The authors declare that the research was conducted in the absence of any commercial or financial relationships that could be construed as a potential conflict of interest.

## Publisher’s Note

All claims expressed in this article are solely those of the authors and do not necessarily represent those of their affiliated organizations, or those of the publisher, the editors and the reviewers. Any product that may be evaluated in this article, or claim that may be made by its manufacturer, is not guaranteed or endorsed by the publisher.

## Abbreviations

ED, erectile dysfunction; AVSS, audiovisual sexual stimulation; NPTR, nocturnal penile tumescence and rigidity; AUC, area under the curve; ROC, receiver operator characteristic; IIEF-EF, international Index for erectile dysfunction- erectile function domain; PDE-5i, phosphodiesterase-5 inhibitors; DOEE, duration of erectile episodes; DOEE60, duration of erectile episodes over 60%; AER, average event rigidity; TOEE60, times of erectile episodes over 60%; TOTT, times of total tumescence.
