# Supplementary material for: Selecting an Individualized Treatment Approach: The Predictive Value of Erotic Stimulation and Nocturnal Erections for Efficacy of Tadalafil and Cure in Patients With Erectile Dysfunction
Source: Front Endocrinol (Lausanne). 2022 Jun 29;13:915025. doi: 10.3389/fendo.2022.915025 (PMC9276996; doi:10.3389/fendo.2022.915025)
Supplement: Supplementary file 1 [file DataSheet_1.doc]

**Table S1. Comparison between tadalafil responders and non-responders via AVSS**

Data are shown as median (interquartile range). ∆Tumescence = (increased or maximum tumescence - minimum tumescence)/minimum tumescence.

| **Parameters** | **tadalafil responders (n=184)** | **tadalafil non-responders (n=42)** | **P-value** |
| --- | --- | --- | --- |
| DOEE (min) | 5.00(16.50) | 0(0) | P < 0.001 |
| DOEE60 (min) | 0(6.38) | 0(0) | P < 0.001 |
| AER of tip (%) | 5.00(51.00) | 0(0) | P < 0.001 |
| AER of base (%) | 9.50(63.00) | 0(0) | P < 0.001 |
| ∆Tumescence of tip (%) | 14.74(28.03) | 0(0) | P < 0.001 |
| ∆Tumescence of base (%) | 18.01(29.29) | 0(0) | P < 0.001 |

**Table S2. Comparison between tadalafil responders and non-responders via NPTR**

Data are shown as median (interquartile range). ∆Tumescence = (increased or maximum tumescence - minimum tumescence)/minimum tumescence.

| **Parameters** | **tadalafil responders (n=158)** | **tadalafil non-responders (n=46)** | **P-value** |
| --- | --- | --- | --- |
| DOEE (min) | 66.13(90) | 33.10(64.88) | P < 0.001 |
| DOEE60 (min) | 26.50(46) | 0.50(11.63) | P < 0.001 |
| TOEE60 | 1.00(2.00) | 0(0) | P < 0.001 |
| TOTT | 3.00(3.00) | 1.50(2.25) | P < 0.001 |
| AER of tip (%) | 53.00(37.25) | 7.5(32.25) | P < 0.001 |
| AER of base (%) | 67.00(40.75) | 14.50(50.25) | P < 0.001 |
| ∆Tumescence of tip (%) | 29.84(20.96) | 16.13(20.94) | P < 0.001 |
| ∆Tumescence of base (%) | 32.52(11.53) | 25.50(18.78) | P < 0.001 |

**Table S3. Comparison between cured patients and uncured patients via AVSS**

| **Parameters** | **Cured patients (n=81)** | **uncured patients (n=145)** | **P-value** |
| --- | --- | --- | --- |
| DOEE (min) | 14.50(18.88) | 0(4.00) | P < 0.001 |
| DOEE60 (min) | 4.50(14.25) | 0(0) | P < 0.001 |
| AER of tip (%) | 45.00(48.00) | 0(0.50) | P < 0.001 |
| AER of base (%) | 49.00(58.00) | 0(3.00) | P < 0.001 |
| ∆Tumescence of tip (%) | 24.24(17.11) | 0(14.09) | P < 0.001 |
| ∆Tumescence of base (%) | 24.14(16.16) | 0(18.38) | P < 0.001 |

Data are shown as median (interquartile range). ∆Tumescence = (increased or maximum tumescence - minimum tumescence)/minimum tumescence.

**Table S4. Comparison between cured patients and uncured patients via NPTR**

| **Parameters** | **Cured patients (n=84)** | **uncured patients (n=120)** | **P-value** |
| --- | --- | --- | --- |
| DOEE (min) | 73.50(88.25) | 43.38(77.87) | P < 0.001 |
| DOEE60 (min) | 32.50(45.00) | 10.25(28.87) | P < 0.001 |
| TOEE60 | 1.00(2.00) | 0(1.00) | P < 0.001 |
| TOTT | 3.00(3.00) | 2.00(3.00) | P =0.001 |
| AER of tip (%) | 60.50(29.75) | 31.00(50.75) | P < 0.001 |
| AER of base (%) | 72.00(33.75) | 42.50(67.75) | P < 0.001 |
| ∆Tumescence of tip (%) | 32.09(17.39) | 22.05(22.91) | P < 0.001 |
| ∆Tumescence of base (%) | 33.98(10.58) | 30.15(14.35) | P =0.01 |

Data are shown as median (interquartile range). ∆Tumescence = (increased or maximum tumescence - minimum tumescence)/minimum tumescence.
